# Systematic molecular analysis of hemophilia A patients from Colombia

**DOI:** 10.1590/1678-4685-GMB-2017-0072

**Published:** 2018-11-14

**Authors:** Luz Karime Yunis, Adriana Linares, Edgar Cabrera, Juan J. Yunis

**Affiliations:** ^1^ Grupo de Patología Molecular, Universidad Nacional de Colombia, Bogotá, D.C., Colombia; ^2^ Fundación Hospital de la Misericordia, Bogotá, D.C., Colombia; ^3^ Grupo de Onco-Hematología Pediátrica, Departamento de Pediatría, Facultad de Medicina, Universidad Nacional de Colombia, Bogotá, D.C., Colombia; ^4^ Programa de Hemofilia, Clínica Infantil Colsubsidio. Bogotá, D.C., Colombia; ^5^ Departamento de Patología, Facultad de Medicina e Instituto de Genética, Universidad Nacional de Colombia, Bogotá, D.C., Colombia; ^6^ Instituto de Genética, Servicios Médicos Yunis Turbay y Cía S.A.S. Bogotá, D.C., Colombia

**Keywords:** Hemophilia A, Factor VIII, IS-PCR, HRM, Colombia

## Abstract

Hemophilia A (HA) is an X-linked recessive disorder and the second most common coagulation disorder with an incidence of 1 in 5,000 live born males. Worldwide, there are 178,500 affected individuals, 60% with the severe form of the disease. Intron 22 and 1 inversions (Inv22 and Inv1) are the most frequent molecular alterations found in severe HA patients with a frequency of 45-50% and 0.5-5%, respectively. We have implemented a systematic cost-effective strategy for the identification of the molecular alteration in HA patients using Inverse shifting-PCR for Inv22 and Inv1, followed by the analysis of the *F8* gene coding region by means of high resolution melting (HRM) PCR and Sanger sequencing in Inv22 and Inv1 negative patients. A total of 33 male HA patients and 6 women were analyzed. Inversion 22 was detected in 14/33 male patients (42.4%), 3/33 (9.1%) had Inv1, 3/33 (9.1%) had large structural variants, and 11/33 (33.3%) single nucleotide/ small frameshift variants. No genetic variant was found in 2/33 patients (6%). With this systematic approach we detected pathogenic variants in 31 out of 33 male affected individuals (94%) tested for the first time.in a cohort of patients from Colombia.

## Introduction

Hemophilia A (HA) is an X-linked recessive disorder and the second most common coagulation disorder with an incidence of 1 in 5,000 live born males (Hoyer, 1994). Worldwide, there are approximately 178,500 affected individuals with HA, and of these, 60% have the severe form of the disease, followed by mild (5-30%), and moderate cases 15% (Savage, 2014).

Different genomic variants are responsible for different HA phenotypes (Higuchi, *et al.*, 1991a,b; Antonarakis, 1995; Antonarakis, *et al.*, 1995; Ahmed, *et al.*, 2005; Albanez, *et al.*, 2011; Rallapalli *et al.*, 2013). Overall, missense variants are responsible for near 50% of all cases of HA. However, Intron 22 and intron 1 inversions (Inv22 and Inv1) are the most frequent molecular alterations found in severe HA patients with a frequency of 45-50% and 0.5-5%, respectively (Woods-Samuels, *et al.*, 1991; Lakich, *et al.*, 1993). Individuals with Inv22 and Inv1, deletions and nonsense mutations, usually have the severe form of the disease and an increased risk for developing inhibitors during treatment (Brackmann *et al.*, 1996, 2000; Astermark *et al.*, 2005, 2013; Abbonizio *et al.*, 2014). In recent years, high resolution melting analysis (HRM) has been used to screen genomic segments to detect regions that could harbor nucleotide variants in different diseases, including HA (Ririe *et al.*, 1997; Lin *et al.*, 2008).

In Colombia thus far, no molecular genotyping services for HA are available. In the present report, we have used a systematic approach to characterize HA variants in a cohort of patients from Bogotá, Colombia. First, Inv22 and Inv1 were analyzed by inverse-shifting PCR (IS-PCR) (Rossetti *et al.*, 2004, 2005, 2008, 2011; Radic *et al.*, 2009). Negative samples for Inv22 and Inv1 were amplified for all 26 exons by HRM (52 PCR-HRM reactions) (Ririe *et al.*, 1997; Lin *et al.*, 2008), followed by DNA Sanger sequencing of altered HRM melting curves (Sanger *et al.*, 1977). Microarray analysis was used in selected cases to further characterize large deletions. With this approach, we were able to detect pathogenic variants in 94% of our patients for the first time in Colombia, with the identification of three new variants.

## Materials and Methods

### Patients

Approval by the ethics committees of all institutions was obtained. A total of 33 HA male patients (27 severe, 5 moderate, and 1 mild HA) and 6 females (patient’s mothers) were included (Table 1). After signing the informed consent, patients had blood samples collected in EDTA tubes for DNA isolation. Additional information regarding disease clinical course, as well as treatment related information was collected.

**Table 1:** Clinical and demographic data of hemophilia A patients from Colombia.

ID	Sex	Severity	Age (years)	FVIII:C	Inhibitors	Family History of HA	Arthropathy*	Family relationships^
HA-01	Male	Severe	8	< 1	NO	NO	NO	
HA-02	Male	Severe	10	0.4	NO	YES	YES	
HA-03	Male	Severe	6	0.5	NO	YES	NO	
HA-04	Male	Severe	5	0.3	NO	NO	NO	
HA-05	Male	Severe	17	0.3	NO	YES	YES	
HA-06	Male	Severe	3	0.7	YES	NO	NO	
HA-07	Male	Severe	7	< 1	YES	NO	YES	
HA-08	Male	Severe	2	0.6	YES	NO	NO	
HA-09	Male	Severe	5	0.7	YES	NO	YES	
HA-10	Male	Moderate	14	2.0	NO	NO	NO	
HA-11	Male	Severe	1	0.6	YES	NO	NO	
HA-12	Male	Moderate	10	1.8	NO	YES	YES	
HA-13	Male	Severe	17	0.8	NO	NO	YES	
HA-14	Male	Severe	18	0.8	YES	YES	YES	
HA-16	Male	Severe	14	0.5	YES	NO	YES	
HA-17	Male	Moderate	13	3.0	NO	YES	NO	
HA-18	Male	Severe	0.5	0.4	NO	YES	NO	
HA-19	Male	Severe	1	< 1	NO	YES	NO	
HA-20	Male	Mild	4	12.0	NO	YES	NO	
HA-22	Female	Carrier	20	70.0	ND	YES	NO	HA-20 Mother
HA-23	Female	Carrier	28	118.0	ND	NO	NO	HA-07 Mother
HA-24	Male	Severe	10	0.5	NO	NO	YES	
HA-25	Male	Severe	5	< 1	NO	NO	NO	
HA-27	Male	Severe	7	0.4	NO	YES	NO	
HA-28	Female	Carrier	37	34.0	NO	YES	NO	HA-27 Mother
HA-29	Male	Severe	2	0.4	NO	YES	NO	
HA-31	Male	Severe	16	0.7	YES	NO	YES	
HA-32	Male	Moderate	14	3.7	NO	NO	YES	
HA-33	Male	Severe	1	0.7	NO	NO	NO	
HA-35	Male	Severe	1	0.5	NO	NO	NO	
HA-36	Male	Severe	0.5	0.4	NO	NO	NO	
HA-37	Male	Severe	1	0.2	NO	NO	YES	
HA-38	Female	Carrier	22	ND	ND	NO	NO	HA-06 Mother
HA-39	Female	Carrier	23	ND	ND	NO	NO	HA-33 Mother
HA-40	Male	Moderate	10	3	NO	NO	NO	
HA-41	Male	Severe	14	0.7	NO	YES	YES	
HA-42	Male	Severe	19	< 1	YES	NO	YES	
HA-43	Male	Severe	11	0.7	NO	NO	YES	
HA-45	Female	Carrier	37	ND	ND	NO	NO	HA-16 Mother

ND, no data, Actual inhibitors or history of inhibitors; * Actual arthropathy or history of arthropathy; ^ Familial relationships between individuals included in this study.

### DNA extraction

DNA was obtained from 200 μL of whole blood using the DNA Blood Mini kit Qiagen, following manufacturer’s recommendation (Qiagen, Hilden Germany). DNA purity and concentration was determined in a Nanodrop 2000 spectrophotometer (Nanodrop, Wilmington, DE).

### Inverse shifting PCR (intron 1 and intron 22 inversion analysis)

Intron 1 and 22 inversions were determined by inverse shifting PCR as described previously (Rossetti *et al.*, 2008). Briefly, 2 μg of genomic DNA was digested with 20 U of *Bcl*I restriction enzyme (Thermo Fisher Scientific, Massachusetts) for 4 h in a 50 μL volume. Digested DNA was purified using Centricon 100 concentrators (Amicon, MA). Self-ligation was carried out in a 400 μL reaction mix containing 3 U of T4 DNA ligase (Thermo Fisher Scientific, MA) at 15 °C for 12–14 h. Self-ligated circles were purified using Centricon 100 concentrators (Amicon) and adjusted to a final volume of 100 μL. For intron 1 inversion analysis, 3 μL was used for PCR amplification, while 6 μL was used for intron 22 inversions. PCR amplifications were carried out in a 25 μL reaction mix with 0.6 μM of each primer: 200 μM dNTP, 1.5 mM MgCl_2_, and 0.5 U GoTaq Flexi DNA polymerase (Promega Corporation, WI) in a C100 BioRad thermal cycler with a protocol of 94 °C for 2 min followed by 30 cycles of 94 °C for 30 s, 56 °C for 1 min and 72 °C for 1.5 min, terminating with a final extension step of 5 min at 72 °C.

Amplified products were resolved in 2% Nusieve 3:1 agarose (Lonza, MD) in 1X SO buffer and visualized using SyberSafe stain (Invitrogen, CA).

### High resolution melting analysis

High resolution melting (HRM) analysis was carried out using Precision Melt Supermix (BioRad, CA) and analyzed in a 96 CFX real time system (BioRad), using primers covering all 26 exons of the *F8* gene, as described previously (Lin *et al.*, 2008). Briefly, 2 μL of genomic DNA (12.5 ng/μL) were used for each reaction containing 5 μL of 2x Precision Melt Supermix, 2 μL primer mixture (10 pmol/μL each), and 1 μL distilled water. An initial denaturing step at 95 °C for 2 min was followed by 45 cycles of 95 °C for 10 s, 53 °C for 30 s and plate reading 72 °C for 30 s. followed by HRM with 1 cycle of 95 °C for 30 s, 1 cycle at 60 °C for 1 min, 1 cycle at 65-95 °C (10 s/step, slope 0.2 °C/s) and plate reading. HRM analysis was done with the Precision Melt analysis software (melt curve sensitivity set at 50, Tm difference threshold of 0.2 °C)

### Microarray analysis

A total of 250 ng of genomic DNA was used for CytoScan 750 microarray (Affymetrix, CA) analysis. Briefly, genomic DNA was digested with *Nsp*1 at 37 °C for 2 h. The digested DNA was purified and ligated to primer-adapters at 16 °C for 3 h, followed by PCR amplification. Amplified DNA was purified and digested with DNAseIat 37 °C for 35 min. Digested DNA was biotin-labeled for 4 h at 37 °C, hybridized to CytoScan 750K microarrays at 50 °C for 18 h and washed to remove unbound sample. Samples were read with AGCC console and ChAS 3.1 (Chromosome Analysis Suite) from Affymetrix.

### DNA sequencing

Samples with different HRM curves were sequenced using the 3.1 BigDye terminators cycle sequencing kit (Applied Biosystems, CA), following manufacturer’s recommendations and analyzed in a 3500 ABI genetic analyzer (Applied Biosystems). DNA sequences were compared to the *F8* gene reference sequence NM_000132.3 using SeqScape V5.4. (Applied Biosystems). Briefly, samples were amplified in a C1000 BioRad thermal cycler in 50 μL volume reactions containing 10 μL *Taq* polymerase buffer, 5 μL primer mix (10 pmol/μL each), 4 μL dNTP (10 nM each), 2 mM MgCl_2_, and 1.25 μLGoTaq DNA Polymerase (Promega, WI) and 5 μL of genomic DNA (12.5 ng/μL). After an initial denaturation step at 95 °C for 2 min, 35 cycles of 95 °C for 10 s, 53 °C for 30 s and 72 °C for 30 s were run, followed by a final extension step of 5 min at 72 °C, PCR-amplified products were purified using PureLink quick PCR purification kit (Invitrogen) following manufacturer’s recommendations. Purified fragments were quantified in a Nanodrop 2000 system and 10 ng of PCR purified fragments were used for BigDye terminator sequencing. Mutation nomenclature was according to the Human Genome Variation Society (HGVS) (den Dunnen *et al.*, 2016)

### Software prediction analysis

To determine the pathogenicity of new identified missense variants, different functional prediction softwares were used, such as POLYPHEN-2, PROVEAN, Mutation Taster, and PhD-SNP (Capriotti *et al.*, 2006; Adzhubei *et al.*, 2010; Choi *et al.*, 2012; Schwarz *et al.*, 2014).

### Statistical analysis

A 2x2 cross-tab was used to analyze the associations between Inv22/inhibitor development and Inv22/ arthropathy by Fisher’s Exact test.

## Results

### Intron 22 and intron 1 inversions

Intron 22 inversion was detected in 14 out of 33 (42.4%) unrelated cases. Twelve samples were type I Inv22 and 2 samples type II Inv22. Three out of 33 samples (9.1%) were positive for Inv1 (Supplementary Figure S1). Two patients’ mothers were positive for Inv22 and an additional mother was positive for Inv1.

### HRM analysis

Samples negative for Inv22 and Inv1 were analyzed by HRM analysis (16 out of 33 patients (48.4%), and 3 mothers). Three samples (HA-07, HA-11, and HA-13) showed lack of amplification for one or more *F8* exons. One of the samples (HA-07), failed to amplify for exons 1 through 14. To verify this result, the same sample was amplified from separate DNA isolates, showing the same result. The patient’s mother (HA-23) showed amplification for all exons. Sample HA-13 failed to amplify exon 13 on separate occasions. Sample HA-11 failed to amplify exon 26. In all cases, poor DNA quality was excluded as the reason for failing PCR amplification since other exons were amplified in parallel simultaneously. In addition, other samples used as controls for amplifications yielded the expected PCR fragment. These results indicated that 3 out of 33 samples had large deletions (9.1%). Of the remaining samples, on average, HRM analysis showed different HRM curves in 3 to 4 fragments. Therefore, DNA sequencing was carried out on those fragments in order to detect the pathogenic variant present in each sample (see below).

### Microarray analysis

Microarray analysis was carried out in those three samples that showed deletions by HRM analysis, as well as in the HA-07 mother. Sample HA-07 showed a large deletion involving exons 1 through 14 of 223,573 bp (arr[hg19] Xq28 (154153703-154377276)x0) that compromised 5 genes: *F8*, *FUNDC2, MTCP1NB, MTCP1*, and *BRCC3*. His mother had the same deletion as a heterozygous (arr[hg19] Xq28 (154153703-154377276)x1) (Supplementary Figure S2). Patient HA-13 had a 17,979 bp deletion that involves part of intron 12, exon13 and intron 13 (arr[hg19] Xq28 (154163600-154181579)x0). Patient HA-11 microarray did not show any deletion in the microarray analysis, even when the filter was lowered to a 1 kb size (Supplementary Figure S2).

### DNA sequencing

Initially, pathogenic variants were identified in nine of the 13 unrelated patients analyzed by HRM (Table 2, Supplementary Figure S3). Missense variants were present in seven samples, a nonsense variant in one sample, and a small frameshift deletion in one sample (Table 2). With this approach we did not identify the pathogenic variant in four patients. We sequenced the entire coding region with the exception of exon 14 in these four patients. Two additional pathogenic variants were identified: c.399T > A; p.Y133* in HA-35 and c.209-212delTTGT; p.F70* in HA-36, increasing the number of identified variants to 11 out of 13 unrelated patients. Most of these variants have been reported previously in hemophilia A databases: CHAMP CDC (https://www.cdc.gov/ncbddd/hemophilia/champs), Hemobase (http://www.hemobase.com), Factor VIII Variant Database (http://www.factorviii-db.org), and the literature, with the exception of three variants, two in severe HA c.262A > G, p.M88V, c.399T > A; p.Y133*, and another in mild HA c.5666A > G; p.Q1889R (Table 3, Supplementary Figure S3).

**Table 2:** Microarray and sequencing genetic variants identified in hemophilia A patients from Colombia.

ID	Genetic variant*	Amino acid substitution	Mutation type	Exon /Intron	FVIII:C	Severity HA	Reported
HA-07	Del exon 1_14	-	Large deletion	1 _14	< 1	Severe	Yes
HA-10	c.1957C > T	p.V653M	Missense	13	2	Moderate	Yes
HA-11	Del exon 26	-	Large deletion	26	0.6	Severe	Yes
HA-13	Del exon 13	-	Large deletion	13	0.8	Severe	Yes
HA-17	c.1292T > C	p.L431S	Missense	9	3	Moderate	Yes
HA-18	c.274G > T	p.G92C	Missense	3	0.4	Severe	Yes
HA-20	c.5666A > G	p.Q1889R	Missense	17	12	Mild	No
HA-24	c.5953C > T	p.R1985*	Nonsense	18	0.5	Severe	Yes
HA-32	c.2095A > G	p.M699V	Missense	13	3.7	Moderate	Yes
HA-33	c.207_208delGT	p.F207Lfs*12	Small deletion/Frameshift	2	0.7	Severe	Yes
HA-35	c.399T > A	p.Y133*	Nonsense	4	0.5	Severe	No
HA-36	c.209_212delTTGT	p.F70*	Small deletion/Frameshift	2	0.4	Severe	Yes
HA-37	c.262A > G	p.M88V	Missense	2	0.2	Severe	No
HA-40	c.1892A > G	p.N631S	Missense	12	3	Moderate	Yes

* the mutation numbering was according to the complete mRNA sequence to keep consistency with other published results; the amino acid numbering is given for the mature processed protein.

**Table 3:** Prediction software analysis of new genetic variants identified in Hemophilia A patients from Colombia

ID	Genetic variant* ^	Amino acid substitution	Severity	Polyphen-2	Provean	Mutation Taster	PhD-SNP
HA-37	c.262A > G	p.M88V	Severe	Probably damaging, 1.0 score	Neutral	Disease causing	Disease
HA-20	c.5666A > G	p.Q1889R	Mild	Possibly damaging, 0.798 score	Neutral	Disease causing	Neutral

* the mutation numbering was according to the complete mRNA sequence to keep consistency with other published articles; the amino acid numbering is given for the mature processed protein; ^ mutations not found in Hemophilia A databases CHAMP CDC, Hemobase, Factor VIII Variant Database.

#### Genotype/phenotype correlations

Twenty male samples showed Inv22, Inv1, or large deletions. Nineteen of them showed a severe HA phenotype. One patient (HA-12) had Inv22 with 1.8% FVIII:C. However, he presented a severe phenotype with history of arthropathy at the age of 8 and was receiving prophylaxis at the time (Table 1). The remaining were female carriers (two with Inv22, one Inv1, and one with large deletion). One female showed FVIII activity (FVIII:C 35%), and the remaining had normal FVIII activity.

Eleven samples had either point mutations or small deletions. Of these, seven patients had severe HA phenotype. One of them, HA-33, had a frameshift deletion of two base pairs c.207-208delGT;p.F207Lfs12*, also found in his heterozygous mother (HA-39); HA-24 had a nonsense variant c.5953C > T; p.R1985*, and HA-18 had a missense variant c.274G > T, p.G92C (Table 2). The remaining samples with missense variants had either moderate or mild phenotypes. Thus, a severe phenotype was mainly associated with Inv22, Inv1, large deletions, frameshift, and nonsense variants, as described previously. Missense variants were mainly associated to moderate and mild phenotypes.

HA family history was present in 12/33 patients (36.3%). Arthropathy was found in 16/33 patients (48.4%). History of FVIII inhibitors was present in 11/33 patients (33.3%), similar to other populations (Gouw *et al.*, 2011); there was no association of a particular genetic variant with FVIII inhibitors. Inversion 22 was present in 7 out of the 11 patients with inhibitors positive history (63.3%) compared to 9/26 (34.5%) inhibitors negative history (Fisher’s exact test *p*=0.151). A similar finding has been reported earlier (Oldenburg *et al.*, 2002; Goodeve and Peake, 2003; Mantilla-Capacho *et al.*, 2007; Ghosh and Shetty, 2009; Tantawy *et al.*, 2011; Kreuz and Ettingshausen, 2014).

### Discussion

We used inverse shifting-PCR for Inv22 and Inv1 (Rossetti *et al.*, 2005, 2008, 2011; Radic *et al.*, 2009) followed by HRM analysis to identify *F8* exon fragments that could harbor pathogenic variants, and DNA Sanger sequencing. With this approach, we were able to detect pathogenic variants in 29 out of 33 affected male individuals. Two additional pathogenic variants were identified by sequencing most of the entire coding sequence in two additional patients (31/33, 94%).

As expected, 92.5% severe HA patients had Inv22, Inv1, frameshift, nonsense, and large deletions (25/27). The remaining two severe HA carried missense variants; one patient with a c.274G > T; p.G92C previously reported in the literature (Tuddenham *et al.*, 1994), and another with a c.262A > G;p.M88V not reported before. This variant was classified as a pathogenic variant in three out of four of the prediction analysis softwares used (Capriotti *et al.*, 2006; Adzhubei *et al.*, 2010; Choi *et al.*, 2012; Schwarz *et al.*, 2014) (Tables 2 and 3, Supplementary Figure S3).

Microarray analysis was carried out in all the patients showing lack of amplification for one or more exons by HRM (Supplementary Figure S2). We chose to analyze large deletions by microarray analysis instead of MLPA since microarray analysis can further delineate deletion points, as well as other genetic regions involved. In addition, MLPA analysis for *F8* would have shown what we had already detected in the HRM amplification, since MLPA probes are directed to exon sequences of the gene.

The HA-07 patient and his mother showed a large deletion involving exon 1 through exon 14, with a total of 223,573 bp missing (Supplementary Figure S2). Similar deletions have been reported previously involving exons 1 through 14 (Tuddenham *et al.*, 1994; Kemball-Cook and Tuddenham, 1997; Giannelli *et al.*, 1998; Kemball-Cook *et al.*, 1998; Krebs *et al.*, 2003; Lindvall and Swedenborg, 2006; Margaglione *et al.*, 2008; Rallapalli *et al.*, 2013; Rydz *et al.*, 2013). In this case, the deletions involved four additional genes *FUNDC2, MTCP1NB, MTCP1*, and *BRCC3*, a finding that has not been reported earlier since no microarray analysis had been performed.

Patient HA-13 had a 17,979 bp deletion that involved part of intron 12, exon 13, and intron 13. Patient HA-11 failed to amplify exon 26 in several occasions by HRM. However, CytoScan 750 microarray analysis failed to detect a deletion, even when a 1 kb filter was used. Detailed analysis by CytoScan 750 microarray in this sample showed absence for probes (SNP and CNV markers) covering this exon, thus explaining the lack of detection for this deletion in the microarray (Supplementary Figure S2).

Five patients had a moderate HA phenotype. One of them had Inv22 (FVIII:C 1.8%) and the remaining four single nucleotide missense variants, all of which had been published before: c.1292T > C;p.L431S (HA-17), c.2095A > G;p.M699V (HA-37), c.1892A > G;p.N631S (HA-40), and c.157C > T;p.V653M (HA-10) (Table 2, Supplementary Figure S3).

A missense variant present in one HA mild patient and his heterozygous mother, c.5666A > G; p.Q1889R (12.4% and 70% FVIII:C activity, respectively) had not been published before. Two out of four prediction tools showed this variation as pathogenic (Capriotti *et al.*, 2006; Adzhubei *et al.*, 2010; Choi *et al.*, 2012; Schwarz *et al.*, 2014) (Tables 2 and 3, Supplementary Figure S3).

The frequencies of pathogenic variants found in our study are similar to those described for other populations, including some Latin American studies. In Argentina, Inv22 frequencies have been reported between 39 and 41% (De Brasi *et al.*, 2000, 2003; Rossetti *et al.*, 2004), very similar to Venezuela in severe HA patients (Albanez *et al.*, 2011), while in Mexico, the frequency reported was 45% (Mantilla-Capacho *et al.*, 2007). In Costa Rica, Inv22 was found in 21/34 severe patients (61.7%) while no Inv1 was detected (Salazar-Sanchez *et al.*, 2010). A slightly higher frequency for Inv1 was found in our study (9.1% of severe HA patients), compared to Argentinean patients.

High resolution melting analysis under standard melting parameters allowed us to identify genetic variants by Sanger sequencing in nine of the remaining 13 patients (69%). High resolution melting analysis gave positive results on average in three out of 52 fragments per sample. In four patients (4/33, 12.1%) we were not able to detect any variant with the approach used in the present study. These results could be due to either false negative HRM reactions, or genetic variants that are located outside of the regions tested here. HRM analysis is based on melting curves that are similar but distinguishable from each other, first, by differences in amplicon melting temperature (Tm), and second, by melting curves showing differences in homozygous and heterozygous samples (Ririe *et al.*, 1997). Since only one amplicon is obtained in male HA patients, only differences in melting temperature curves are evaluated in X-linked diseases. Thus, differences in homozygous and heterozygous melt temperature curves are not accounted for.

In this regard, it is interesting that the genetic variant present in the HA-33 patient (a 2 bp deletion) was suspected once the sample from the patient’s mother gave an abnormal melt pattern in exon 2 by HRM analysis that was not detected initially in the patient’s HRM analysis. Thus, it is possible that false negative HRM reactions are accountable for these results. We identified two additional pathogenic variants in two additional patients when we sequenced the entire *F8* coding regions with the exception of exon 14. The remaining two pathogenic variants could lie within exon 14 or within introns, outside of the regions tested here.

Previous studies have shown that 2% of the genetic variants in HA are due to mutations found in non-coding sequences (Hallden *et al.*, 2012; Pezeshkpoor *et al.*, 2013). In addition, a third homologous recombination region has been described in HA (Pezeshkpoor *et al.*, 2012) that was not tested in our study. Thus, further studies are required to identify the pathogenic variants in those two samples.

### Conclusion

For the first time in Colombia we have used a systematic cost-effective molecular approach to detect HA pathogenic variants. This approach was used due to the high cost for methods such as next generation sequencing (NGS) in our health system. With this approach we were able to detect pathogenic variants in 94% of patients (31/33). Also, we identified three new genetic variants not reported previously in the CHAMP CDC, Hemobase, Factor VIII Variant Database, and the literature. Further analysis is underway to identify the genetic variant responsible for the HA severe phenotype in the remaining two patients.

## Supplementary material

The following online material is available for this article:

Figure S1 - Inverse shifting-PCR for Inv22 and Inv1.

Figure S2 - CytoScan 750 (Affymetrix) microarray analysis.

Figure S3 - Hemophilia A BigDye terminators direct sequencing of Colombian HA patients.
