# Supplementary material for: Systematic molecular analysis of hemophilia A patients from Colombia
Source: Genet Mol Biol. 2018 Nov 14;41(4):750–7. doi: 10.1590/1678-4685-GMB-2017-0072 (PMC6415612; doi:10.1590/1678-4685-GMB-2017-0072)
Supplement: Supplementary file 1 [file 1415-4757-GMB-1678-4685-GMB-2017-0072-s001.pdf]

Supplementary Material to: "Systematic molecular analysis of hemophilia A patients from Colombia"

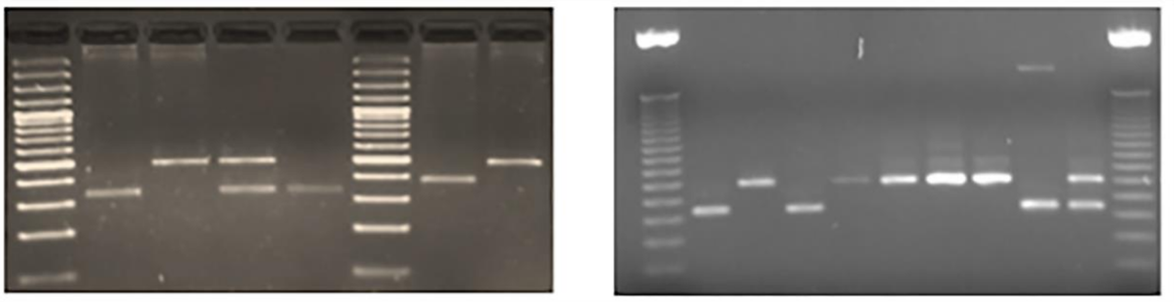

Figure S1 - Inverse shifting-PCR for Inv22 and Inv1. Left: gel electrophoresis in 2% Nusieveagarose gel for intron 22 inversion (Inv22). Ladder 100 bp, lanes 1 and 6. Inv22 type I (333bp) positive samples lanes 2 and 5. Inv22 type 1 carrier female lane 4. Inv22 type II positive (385 bp) sample lane 7. Inv22 negative samples (487 bp), lanes 3 and 8. Right: gel electrophoresis in 2% Nusieveagarose gel for Inv1. 50 bp ladder, lanes 1 and 11. Samples with Inv1 positive (224 bp) reactions (HA-1, 14, 27, 28 (heterozygous carrier) lanes 2, 4,9,10. Samples Inv1 negative (304 bp) lanes 3, 5, 6, 7, 8.
