# Supplementary material for: Systematic molecular analysis of hemophilia A patients from Colombia
Source: Genet Mol Biol. 2018 Nov 14;41(4):750–7. doi: 10.1590/1678-4685-GMB-2017-0072 (PMC6415612; doi:10.1590/1678-4685-GMB-2017-0072)
Supplement: Supplementary file 2 [file 1415-4757-GMB-1678-4685-GMB-2017-0072-s002.pdf]

# Supplementary Material to: "Systematic molecular analysis of hemophilia A patients from Colombia"

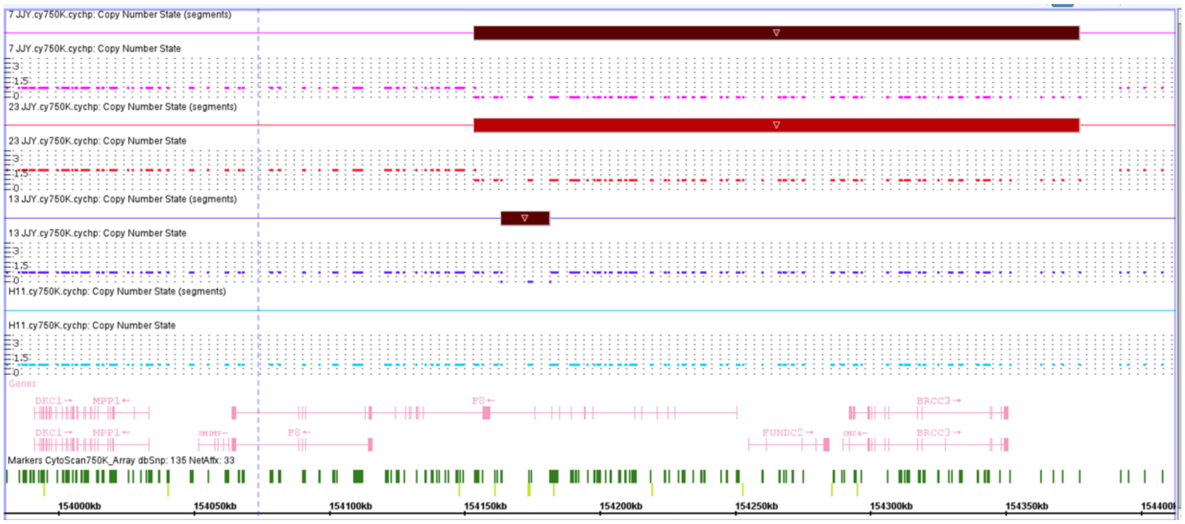


**Figure S2** - CytoScan 750 (Affymetrix) microarray analysis. Two upper signals: Dark red, HA-07 male hemophilic A patient, showing a large deletion involving exon 1 through exon 14, with a total of 223,573 bp (arr[hg19] Xq28 (154153703-154377276)x0) that compromised 5 genes F8, FUNDC2, MTCP1NB, MTCP1, and BRCC3 (genes are represented in pink at the bottom of the figure, vertical bold lines represent F8 gene exons. Notice orientation of F8 gene from right to left). Red, HA-23, mother of HA-07, had the same deletion as a heterozygous (arr[hg19] Xq28 ( 154153703-154377276)x1). Third signal: Dark red, HA-13 patient had a 17,979 bp deletion that involves part of Int12, exon13 and Int13 (arr[hg19] Xq28 (154163600-154181579)x0). Lower Signal: HA-11 patient with exon 26 deletion detected by HRM. However, microarray failed to identify deletion due to lack of probe markers covering exon 26. Vertical green lines are probes present in the array. Notice that there are no probes covering exon 26.
