# Supplementary material for: Systematic molecular analysis of hemophilia A patients from Colombia
Source: Genet Mol Biol. 2018 Nov 14;41(4):750–7. doi: 10.1590/1678-4685-GMB-2017-0072 (PMC6415612; doi:10.1590/1678-4685-GMB-2017-0072)
Supplement: Supplementary file 3 [file 1415-4757-GMB-1678-4685-GMB-2017-0072-s003.pdf]

Supplementary Material to: "Systematic molecular analysis of hemophilia A patients from Colombia"

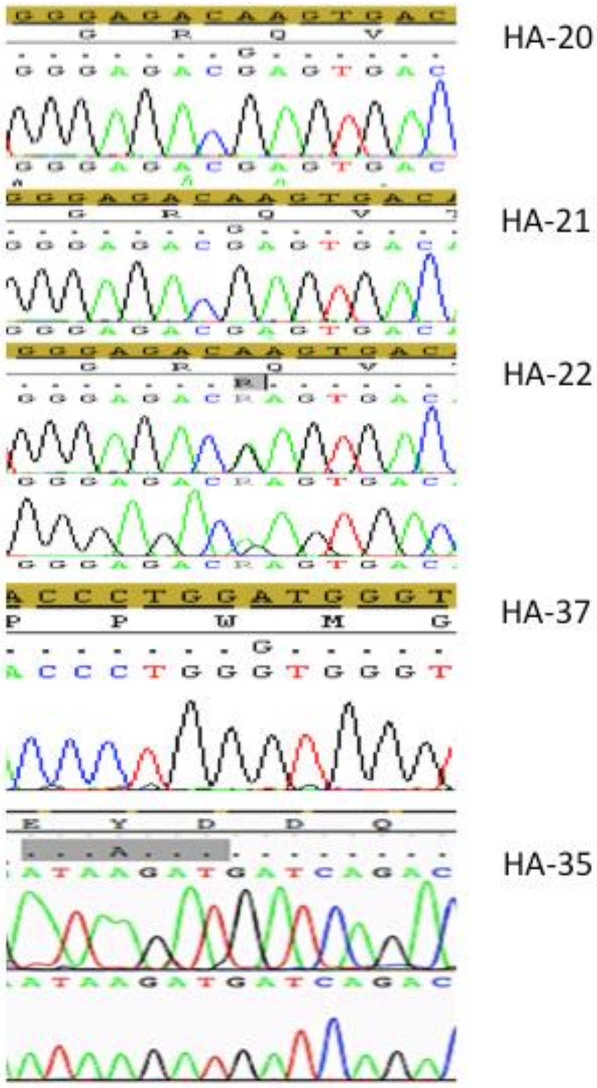

Figure S3 - Hemophilia A BigDye terminators direct sequencing of Colombian HA patients. HA-20, HA-21, c.5666A>G (p.Q1889R), exon 17, HA-22 carrier mother, c.5666A>R (p.Q1889Q,R); HA-37 exon 2, c.262 A>G (p.M88V); HA-35 c.399T>A; p.Y133*.
